# New Learning of Music after Bilateral Medial Temporal Lobe Damage: Evidence from an Amnesic Patient

**DOI:** 10.3389/fnhum.2014.00694

**Published:** 2014-09-03

**Authors:** Jussi Valtonen, Emma Gregory, Barbara Landau, Michael McCloskey

**Affiliations:** ^1^Institute of Behavioural Sciences, University of Helsinki, Helsinki, Finland; ^2^Department of Cognitive Science, Johns Hopkins University, Baltimore, MD, USA

**Keywords:** music performance, learning, memory, hippocampus, brain damage, anterograde amnesia, single-patient study

## Abstract

Damage to the hippocampus impairs the ability to acquire new declarative memories, but not the ability to learn simple motor tasks. An unresolved question is whether hippocampal damage affects learning for music performance, which requires motor processes, but in a cognitively complex context. We studied learning of novel musical pieces by sight-reading in a newly identified amnesic, LSJ, who was a skilled amateur violist prior to contracting herpes simplex encephalitis. LSJ has suffered virtually complete destruction of the hippocampus bilaterally, as well as extensive damage to other medial temporal lobe structures and the left anterior temporal lobe. Because of LSJ’s rare combination of musical training and near-complete hippocampal destruction, her case provides a unique opportunity to investigate the role of the hippocampus for complex motor learning processes specifically related to music performance. Three novel pieces of viola music were composed and closely matched for factors contributing to a piece’s musical complexity. LSJ practiced playing two of the pieces, one in each of the two sessions during the same day. Relative to a third unpracticed control piece, LSJ showed significant pre- to post-training improvement for the two practiced pieces. Learning effects were observed both with detailed analyses of correctly played notes, and with subjective whole-piece performance evaluations by string instrument players. The learning effects were evident immediately after practice and 14 days later. The observed learning stands in sharp contrast to LSJ’s complete lack of awareness that the same pieces were being presented repeatedly, and to the profound impairments she exhibits in other learning tasks. Although learning in simple motor tasks has been previously observed in amnesic patients, our results demonstrate that non-hippocampal structures can support complex learning of novel musical sequences for music performance.

## Introduction

Performing music has been described as one of the most demanding forms of skilled serial action human beings are capable of (e.g., Palmer, 1997; Altenmüller and Schneider, 2009). The musician must execute intricate musical sequences expressively under precise timing constraints, following a hierarchically organized rhythmic structure while simultaneously preparing for subsequent notes. Behavioral studies have revealed many important aspects of the cognitive mechanisms that support music performance (Sloboda, 1984, 1985; Palmer and van de Sande, 1993, 1995; Chaffin and Imreh, 1997, 2002; Engel et al., 1997; Palmer, 1997, 2005, 2006; Drake and Palmer, 2000; Finney and Palmer, 2003; Palmer and Pfordresher, 2003; Stewart, 2005; Brodsky et al., 2008; Chaffin et al., 2009; Lehmann and Kopiez, 2009; Snyder, 2009; Simmons, 2012; van Vugt et al., 2012; Verrel et al., 2013). However, relatively few studies have shed light on the neural substrates. Among the reasons for the relative dearth of cognitive neuroscience research on music performance are technical difficulties in neuroimaging complex motor behavior, a lack of animal models, and the scarcity of musically proficient neuropsychological research patients [see Peretz and Zatorre (2005), Zatorre et al. (2007), and Levitin and Tirovolas (2009), for reviews on the cognitive neuroscience of music].

In this article, we address a central question concerning the neural bases for music performance, asking whether the hippocampus is necessary for learning to perform new musical pieces by sight-reading. We studied the learning of novel musical pieces by a newly identified amnesic patient, LSJ, who was a skilled amateur violist prior to suffering virtually complete bilateral destruction of her hippocampus due to herpes encephalitis.

The hippocampus, located within the brain’s medial temporal lobes (MTL), is crucial for the acquisition of new declarative memories – memories that can be voluntarily retrieved (Scoville and Milner, 1957; Eichenbaum, 2000, 2013; Squire and Knowlton, 2000; Corkin, 2002; Insausti et al., 2013). In contrast, it has been argued that “procedural” or “motor” learning relies on structures other than the hippocampus and surrounding MTL areas (Squire et al., 2004; Eichenbaum, 2013; Reber, 2013). Consistent with this contention, a number of studies have reported that amnesic patients, including those with severe hippocampal damage, may show preserved capacities for certain forms of non-declarative learning (Milner, 1962; Corkin, 1968, 2002; Stefanacci et al., 2000; Eichenbaum, 2013). These results raise the possibility that learning to perform new pieces of music may be achievable in the absence of the hippocampus. However, it is not entirely clear that the forms of learning demonstrated by amnesic patients in prior studies are comparable in complexity to learning musical pieces for performance.

Learning to perform a piece of music has at times been equated with “procedural,” “non-declarative,” or “motor” learning (e.g., Crystal et al., 1989; Cowles et al., 2003; Cavaco et al., 2012; Simmons, 2012), implying that music performance recruits only learning processes for which the hippocampus is not critical. However, applying this terminology to music performance may be misleading. Stanley and Krakauer (2013) have recently argued that many motor skills such as music performance involve considerable cognitive complexity not required by the simple motor tasks that define procedural learning. As they point out, the distinction between declarative and procedural (or non-declarative) learning was originally based on studies with amnesic patient HM, who had portions of his hippocampus and surrounding MTL structures surgically removed. HM exhibited wide-ranging and profound impairments in various learning tasks requiring explicit retrieval, but showed improvement through repetition in simple motor tasks such as mirror drawing (Milner, 1962; Corkin, 1968, 2002; Eichenbaum, 2013). In contrast to how these results have often been interpreted, Stanley and Krakauer (2013) contend that what HM acquired in mirror drawing was not a motor skill but improved motor acuity, one component of motor skill. HM gained fine-tuned precision through repetition of explicitly indicated, identical motor movements. Complex motor skills such as music performance require not only motor acuity but also the ability to select the correct actions on the basis of factual knowledge (Stanley and Krakauer, 2013). Consistent with the distinction between gaining motor acuity and improvement in music performance skills, intact learning in simple motor acuity tasks (e.g., ones in which patient HM showed learning) does not guarantee the ability to learn to play a new piece of music (Beatty et al., 1999).

Sight-reading music requires being able to execute novel combinations of musical sequences that the performer has never encountered before. These demands distinguish sight-reading of music from the production of well-rehearsed motions (e.g., Lehmann and Kopiez, 2009). Unlike simple motor tasks such as mirror drawing, sight-reading of new music poses large cognitive demands (Kinsler and Carpenter, 1995; Furneaux and Land, 1999; Palmer, 2006). The separate dimensions of pitch, rhythm, and meter must be extracted from the notation, combined into a single representation for each event and prepared for execution in ordered sequences at a pre-specified rate. In addition, the processed elements must be held in a memory buffer while the rest of the sequence is being prepared (Kinsler and Carpenter, 1995; Palmer, 1997; Lehmann and Kopiez, 2009). Therefore, an essential aspect of what the sight-reader learns through practice with a new piece of music is more efficient mental planning of the ordered events (Palmer and van de Sande, 1993, 1995; Drake and Palmer, 2000; Palmer and Pfordresher, 2003; Palmer, 2006). In skilled musicians, these mental plans include representations both specific for and independent of the motor programs used to execute them (Sloboda, 1984; Palmer and Meyer, 2000; Meyer and Palmer, 2003; Palmer, 2005, 2006; Brodsky et al., 2008).

In all likelihood, both hippocampal and non-hippocampal structures normally support the complex processes involved in learning to perform a novel piece. Several lines of indirect evidence suggest that the hippocampus is especially important. First, outside the domain of music, the hippocampus has been shown to be important both for the learning of single items and for the ability to form associations between previously unrelated items (Henke et al., 1999; Eichenbaum, 2000; Stark et al., 2002; O’Kane et al., 2004; Squire et al., 2004; Schapiro et al., 2014). As musical pieces are composed from a limited number of basic elements, the ability to form associations between items should be of central importance in learning any new piece of music. Second, some have suggested that the hippocampus plays a special role in memory under conditions that require combining information from multiple sources (Squire and Knowlton, 2000; Nadel and Peterson, 2013); music performance requires integrating separate aspects of musical information related to pitch, rhythm, and meter from visual, auditory, and tactile sensory modalities. Third, music perception studies have shown that damage to the MTL region impairs the ability to learn new melodies in recognition tasks (Wilson and Saling, 2008), and fMRI studies indicate that the hippocampus is recruited in memory tasks involving recognition of novel melodies (Watanabe et al., 2008).

Additional evidence that the hippocampus is important for learning music comes from neuroimaging studies demonstrating that the hippocampus is engaged when complex temporal sequences are learned during motor performance. Specifically, evidence comes from the serial reaction time task (SRT task; Nissen and Bullemer, 1987; Janata and Grafton, 2003; Schendan et al., 2003), a temporal sequence learning paradigm thought to model some of the cognitive and motor aspects related to learning through music performance, albeit in a highly simplified form. In the SRT task, a visual cue appears in one of several spatial locations, and participants are instructed to press the corresponding button as quickly as possible. With practice, participants become faster in responding to repeated sequences than to random ones, even when they are unaware of any repeating patterns. Neuroimaging studies with neurologically intact subjects indicate that the MTL and hippocampus are engaged when complex SRT sequences are learned (Schendan et al., 2003; Robertson, 2007), indirectly suggesting that the hippocampus may also be recruited when new music is learned through performance. Hippocampal activation has also been observed during implicit sequence learning in studies of serial color matching (Gheysen et al., 2010) and oculomotor sequence learning (Albouy et al., 2008). One suggestion is that the hippocampus supports the learning of higher-order temporal associations in practiced sequences (Schendan et al., 2003; Albouy et al., 2008), a function that could be crucial for learning to perform a new piece of music.

Therefore, a relevant similarity between the SRT task and music performance could be that learning complex sequences requires forming higher-order associations among individual elements in both contexts. In terms of neural mechanisms, the MTL has been shown to be involved in learning when information about higher- but not lower-order patterns is acquired (Schendan et al., 2003; Robertson, 2007). In addition, studies with amnesic patients have shown preserved learning in simple forms of the SRT task (Reber and Squire, 1994, 1998), but other studies show impaired performance when the learning of higher-order associations is required (Curran, 1997). These group studies have included some patients with MTL damage, but as the extent of hippocampal damage is unreported and the results are considered at a group level only, the implications are not clear for the role of the hippocampus in learning. Together, however, the findings raise the possibility that learning of new music in the absence of the hippocampus may be unattainable. On the other hand, music performance by sight-reading differs from motor sequence learning tasks in various ways. For example, unlike the SRT task, music performance relies on a large body of previously acquired factual knowledge about musical rules, following a hierarchically organized rhythmic structure and making complex choices about fingerings and hand positions. Conceivably, such a wide range of previously obtained complex abilities could support learning in music performance in a way that is not possible in an SRT-type task. In addition, and perhaps not trivially, the music itself could matter for learning; in music performance, one produces esthetically and emotionally meaningful sounds absent from the SRT task.

Whether non-hippocampal structures alone can support any aspects of new music learning in a performance context is currently not known. Just two studies of brain-damaged individuals have examined whether the hippocampus is necessary for learning in music performance. Cowles et al. (2003) described SL, a patient presumed to have Alzheimer’s disease, whose brain damage included bilateral atrophy in the MTL. SL was taught to play a new song on the violin from sheet music, which he was able to accomplish in two training sessions. Cowles et al. (2003) concluded that the learning of new music does not depend on an intact hippocampus. However, the extent of the patient’s hippocampal damage is unreported, leaving unclear whether the observed learning was (at least partly) supported by remaining hippocampal tissue. In another study, Cavaco et al. (2012) studied a more severely amnesic patient, SZ. This amateur saxophonist had sustained MTL damage, including bilateral damage to the hippocampus, but was able to sight-read music and play in an orchestra. Cavaco et al. (2012) tested SZ’s music performance on 11 target songs before and after biweekly practice with the orchestra over a period of 100 days. They found modest improvement for two of the five dimensions on which SZ’s playing was rated: overall sight-reading accuracy and notes awareness (i.e., the correct identification of written notes and ability to correct one’s errors). The magnetic resonance imaging (MRI) images for SZ indicate that at least some hippocampal tissue remained (see Cavaco et al., 2012). Hence, as in the Cowles et al. (2003) study, SZ’s learning may have been supported by remaining hippocampal tissue. Further, no objective evaluations of the patient’s performances before and after learning were reported by Cavaco et al. [or by Cowles et al. (2003)]. This makes it difficult to estimate the initial difficulty of the material for the patients, or to evaluate the learning trajectory by objective criteria. In addition, although the Cavaco et al. (2012) study is noteworthy for its ecologically valid setting and materials, neither the target nor control songs were pre-designed to control for any of the various factors possibly affecting performance, such as piece length, note type, key signature, or hand position changes^1^.

In sum, it remains an open question whether the hippocampus is necessary for learning to perform a new piece of music, or whether at least some music performance learning can be supported by non-hippocampal structures alone. To investigate this issue, we studied the learning of novel musical pieces through sight-reading in a newly identified amnesic patient, LSJ. LSJ suffered near-complete destruction of her hippocampus bilaterally as a result of herpes encephalitis, and consequently exhibits extremely severe anterograde and retrograde amnesia. Prior to her illness, LSJ was a skilled amateur violist, and informal observations revealed that she could still play the viola by sight-reading at an advanced level. However, her anterograde amnesia is so severe that merely moments after performing a piece from sheet music, she shows no recollection of having encountered the piece before. We investigated whether LSJ could nevertheless show learning for new pieces of music, as revealed by improved performance resulting from practice.

Our study offers new evidence for three important reasons. First, unlike the patients in previous studies, LSJ has virtually no intact hippocampal tissue. Hence, any learning observed in her performance could not be attributed to hippocampal structures. Second, we used novel pieces of music that were specially designed to control for various factors affecting musical complexity, allowing for careful comparisons across pieces. Third, we carried out several analyses that provide a clearer basis for conclusions than in previous studies: LSJ’s performance was evaluated before and after practice with a detailed note-by-note analyses and subjective whole-piece performance judgments made by a group of musicians.

Because some authors have argued that the hippocampus plays a critical role in memory consolidation for temporal sequence learning (Albouy et al., 2008, 2013a,b), we also wanted to investigate whether the learning could be retained in the absence of the hippocampus. Therefore, we tested LSJ’s performance not only on the day of practice but also 14 days after practice.

## Materials and Methods

### Case description

LSJ was 62 years old at the time of the study. Prior to contracting herpes simplex encephalitis (HSE) at age 57, she was a successful professional illustrator. Her illustrations appeared in books, magazines, and newspapers, including many New York Times articles and several covers for The New Yorker magazine. She has a Bachelor of Fine Arts degree.

Prior to her illness, LSJ was a skilled amateur violist and played in several chamber groups and orchestras. She received piano lessons from 6 to 11 years of age, violin lessons from ages 10 to 12, and viola lessons from age 12 until a few years after college. She played the viola in her school orchestra in junior high school, in her high school orchestra for 4 years, in chamber music quartets with friends, and in a university orchestra for 2 years. During college, she played the viola in a theater orchestra and completed several college music courses in viola, violin, ensemble, and symphony orchestra. In the early 2000s, only a few years before her illness, she played viola and sang in the chorus of a community orchestra.

**Figure 1:**
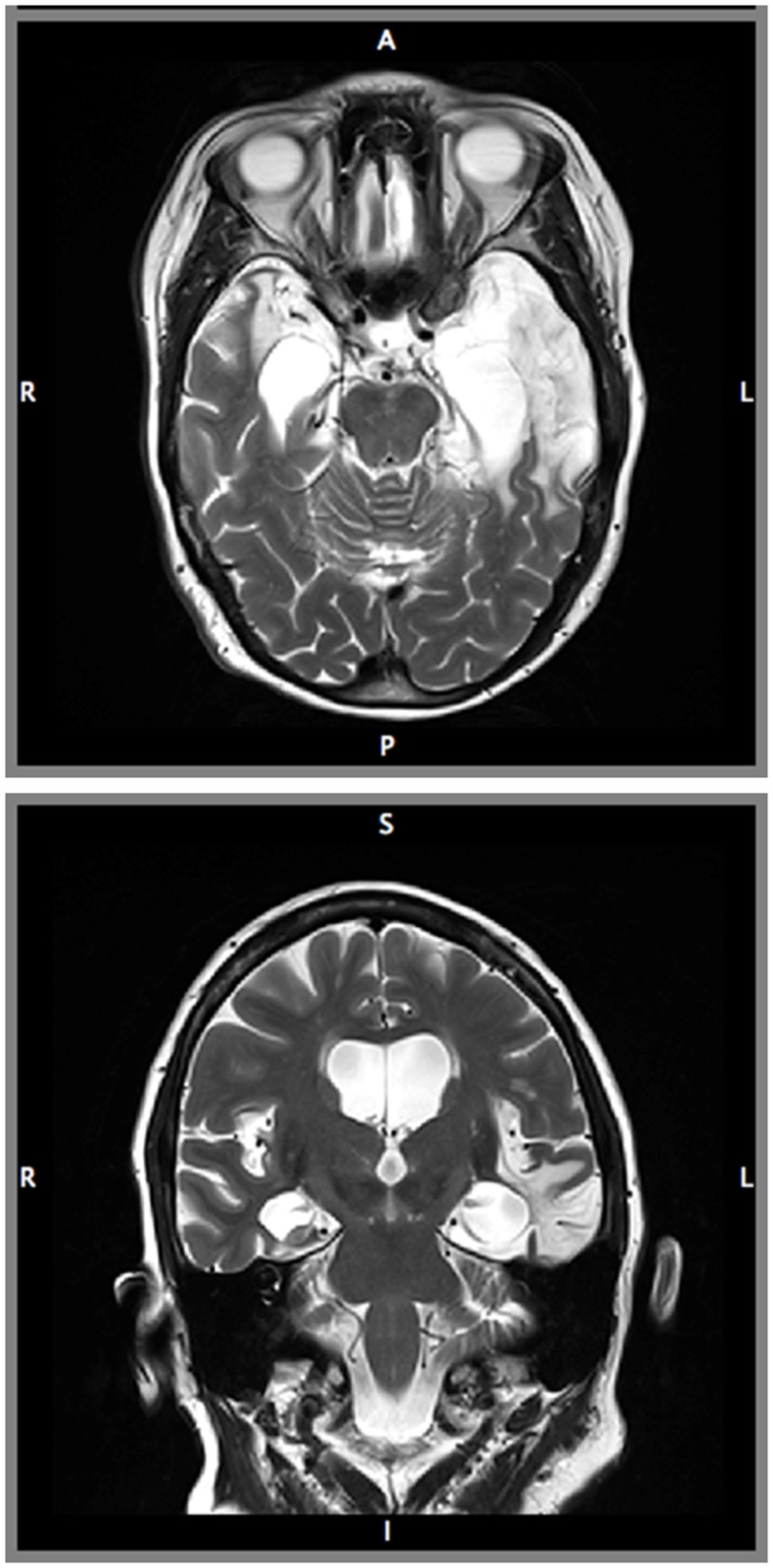
**Magnetic resonance images of patient LSJ’s brain: axial (Top) and coronal (Bottom) view**.

Structural MRI revealed severe bilateral damage to the MTL and anterior temporal damage in the left hemisphere (Figure 1). A volumetric analysis of LSJ’s MTL region (Table 1; Schapiro et al., 2014) showed extensive bilateral damage to the hippocampus, parahippocampal cortex, entorhinal cortex, and perirhinal cortex, as compared to age-matched controls. Most importantly, the analysis demonstrated the near-complete elimination of the hippocampus bilaterally.

**Table 1:** **Remaining brain volume in patient LSJ by MTL region (Schapiro et al., 2014)**.

MTL region	Remaining volume relative to age-matched controls (*N* = 4)	
	Left (%)	Right (%)
Hippocampus	4	0
Parahippocampal cortex	12	62
Entorhinal cortex	0	43
Perirhinal cortex	2	50

LSJ’s general intellectual capabilities are largely spared, and her speech production, comprehension, reading, and visuo-spatial skills are intact or nearly so [see Table 2; for full neuropsychological profile see Gregory et al. (2014)]. On the Wechsler Adult Intelligence Scale IV (Wechsler, 2008), she scored at the 30th percentile. Her single-word reading and spelling were in the normal range, 58th percentile and 55th percentile, respectively, on the Wide Range Achievement Test III (Wilkinson, 1993), and her vocabulary score was at the 63rd percentile on the Peabody Picture Vocabulary Test-Revised (Dunn and Dunn, 1981). On the Boston Naming Test (Kaplan et al., 1983), she scored 49/60, at the low end of the normal range (49–59). In tests of visuo-spatial abilities, her performance was in the normal range on the Visual and Object Space Perception Battery (Warrington and James, 1991), and on the Block Design and Matrix Reasoning subtests on the WAIS-IV.

**Table 2:** **LSJ’s performance on the WAIS-IV, WMS-III, and MBEA [for full neuropsychological test profile see Gregory et al. (2014)]**.

Test	Score	
Wechsler Adult Intelligence Scale IV (WAIS-IV)		
Full scale	92 (30th percentile)	
Verbal comprehension	96 (39th percentile)	
Perceptual reasoning	104 (55th percentile)	
Working memory	83 (13th percentile)	
Processing speed	86 (18th percentile)	
Wechsler Memory Scale III (WMS-III)		
Auditory immediate	56 (0.2 percentile)	Impaired
Visual immediate	57 (0.2 percentile)	Impaired
Immediate memory	47 (<0.1 percentile)	Impaired
Auditory delayed	58 (0.3 percentile)	Impaired
Visual delayed	56 (0.2 percentile)	Impaired
Auditory recognition delayed	55 (0.1 percentile)	Impaired
General memory	47 (<0.1 percentile)	Impaired
Working memory	76 (5 percentile)	Impaired
Montreal Battery of Evaluation of Amusia (MBEA)		
Scale	25	Pass
Contour	25	Pass
Interval	27	Pass
Rhythm	26	Pass
Meter	28	Pass
Incidental	16	Fail

In sharp contrast to her largely preserved general intellectual functions, LSJ presents with extremely profound retrograde and anterograde amnesia (Gregory et al., 2014; Schapiro et al., 2014). Anecdotally, she does not seem to recognize any of our research team, despite having seen us many times; she shows no recollection of tasks she has completed only moments before; and she seems to lose awareness for everyday events very shortly after they have occurred. On the Wechsler Memory Scale III (Wechsler, 1997), she scored below the 0.1 percentile on the General Memory index, with performance severely impaired on all subscales except for working memory, which showed milder impairment (see Table 2). On the Warrington Recognition Memory Test (Warrington, 1984), she performed at chance for both words (26/50) and faces (28/50). Her direct copy of the Rey–Osterrieth figure was normal (34/36), but she scored 0/36 on a recall test after a 10-min delay. Her performance was impaired in tasks requiring statistical learning, the ability to extract regularities in the co-occurrence of items in sequences of shapes, syllables, scenes, or tones (Schapiro et al., 2014).

Thorough interviews with LSJ failed to show memory for even a single specific episode from her life before her illness. For example, she was unable to remember anything from her 10-year marriage including the day she married or was divorced, and even seemed uncertain as to whether she had ever been married. Gregory et al. (2014) found that her retrograde memory impairment extends across not only autobiographical and episodic memory, but also everyday general world knowledge and pre-morbid areas of expertise. Gregory et al. (2014) examined LSJ’s memory for a range of everyday general world knowledge domains, including company names for commercial logos, events associated with everyday songs (e.g., New Year’s with *Auld Lang Syne*), and commonly known facts about sports. LSJ performed far below the level of age- and education-matched controls in both cued recall and forced choice tests. She was also severely impaired relative to controls in tests of visual art and music knowledge, despite her extensive pre-morbid knowledge in those areas. She performed poorly in recalling or selecting the artists of famous paintings (e.g., Monet for *Waterlilies*), and she was unable to name the composer for any of the 61 clips from famous classical pieces (e.g., *Eine Kleine Nachtmusik*). When asked to choose the composer from three alternatives, she performed at chance.

Despite these broad and extensive memory impairments, many of LSJ’s musical abilities appear to be preserved. On the Montreal Battery of Evaluation of Amusia (Peretz et al., 2003), she scored in the normal range on all subtests except the memory test (see Table 2). In a task constructed to assess her knowledge of musical symbols, she exhibited difficulties in verbally naming key signatures (4/26 trials correct) and notes and rests according to their duration (e.g., “a quarter-note,” “a whole rest”; 12/24 and 5/16 trials correct, respectively), but was quite accurate at naming note pitches (88/96 trials correct), clefs (4/4 trials correct), the number of beats designated by note and rest durations (10/12 and 6/8 trials correct, respectively), and the pitches designated as sharps or flats by different key signatures (11/14). We are not able to determine whether her sight-reading or music performance skills are at her pre-morbid level, but when presented with sight-reading material on the viola at an easy to medium-level, she performs fluently and with expression. In performance, she exhibits no difficulty in understanding musical notation, either in the treble or the alto clef.

### Stimuli, design, procedure, and analyses

#### Stimuli

To investigate LSJ’s ability to learn through performance, three novel pieces of viola music were composed (A, B, and C) in a semiclassical style. To make the three pieces as comparable as possible, care was taken to control for various factors that contribute to a piece’s complexity. For example, on a string instrument, a piece that requires frequent changes in hand position is considerably more difficult to sight-read than a piece that can be played throughout in first position. Similarly, it is important to control for the occurrences of specific musical events such as accidentals (sharps or flats outside the piece’s key signature) or double-stops (two notes played simultaneously on two strings), because such events add to the cognitive processing load during sight-reading and often are, as in the case of double-stops, technically more difficult to play than single notes.

Therefore, across the three composed pieces, the following factors were closely matched (see Tables 3 and 4): piece length (both in measures and individual notes), key signature, time signature, note durations, double-stops and their durations, accidentals, clef changes, notes played in each of two clefs (alto and treble), harmonics (notes played by barely touching the string with the left-hand finger), slurs (notes played under a single bow stroke) and type of notes within slurs, notes played using the fourth finger, anticipated string crossings, anticipated hand position changes, and the number of notes played in each of the two anticipated hand positions.

**Table 3:** **Number of notes by duration and clef in the pieces used in the experiment**.

Note type	Piece		
	A	B	C
Dotted quarter notes	1	1	1
Eighth notes	145	145	145
Half notes	15	15	15
Quarter notes	80	78	80
Quarter triplets	6	6	6
Whole notes	1	1	1
Total	248	247	248
Quarter notes in treble clef	8	8	8
Half notes in treble clef	4	4	4
Eighth notes in treble clef	16	16	16
Total number of notes in treble clef	28	28	28
Total number of notes in alto clef	220	219	220
Total	248	247	248

**Table 4:** **Type and number of other musical events matched across pieces in the pieces used in the experiment**.

Musical event	Piece		
	A	B	C
Quarter-note double-stops	4	4	4
Half-note double-stops	6	6	6
Eighth-note double-stops	2	2	2
A# accidentals	1	1	1
D# accidentals	3	3	3
Bb accidentals	2	2	2
Quarter-note slurs	6	6	6
Eighth-note slurs	52	52	52
Harmonics	2	1	1
Notes played with 4th finger	6	6	5
String crossings	119	119	119
Hand position shifts	4	4	4
Notes played in third position	24	24	21

All three pieces were composed so that they could be played on the viola in their entirety in the first and third positions. The number of anticipated position changes was matched by assuming that a violist will play in first position unless forced to move, and by introducing across the three pieces the same number of high notes that cannot be played in first position on the highest string (thereby forcing the performer to move up to third position for these notes). As shown in Table 4, hand position changes were anticipated to occur four times in each piece, with the largest part of the composition assumed to be played in first position.

The number of string crossings and use of the fourth finger were matched by assuming that a violist will only cross strings when necessary and, in a passage of running eighth notes, will use the fourth finger instead of playing an open string. It was also assumed that the violist will use each finger to play only a certain note in a given position, and elect to use the fourth finger only for the notes assigned to it in that position.

Although homogenous in all these respects, the three pieces sounded distinct. Within the same key signature, Piece A focused harmonically on the D major pentatonic, Piece B on D major, and Piece C on B minor (the relative minor of D major, sharing the same key signature). The pieces were all composed to conform to general conventions of Western classical tonal music. That is, they contained no deliberate violations of typical expectations an experienced performer would have of tonal or harmonic structure, meter, phrasing, or fingerings (for the sheet music and computer software performances of all three pieces, see Supplementary Material).

#### Design and procedure

**Figure 2:**
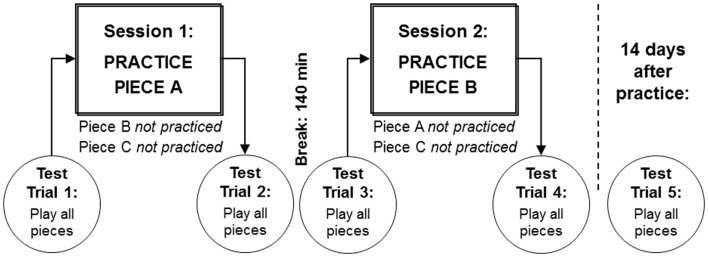
**Two practice sessions were conducted during the same day, in which LSJ practiced two pieces on the viola**. Piece A was practiced in Session 1, and Piece B in Session 2. LSJ’s performance on all pieces was evaluated at a tempo of 144 bpm in test trials before and after each practice session, and 14 days after practice.

LSJ practiced playing two of the pieces on the viola in two different sessions during the same day: Piece A was practiced in Session 1, and Piece B in Session 2 (see Figure 2). Piece C was not practiced and therefore served as a control for the other two.

During each practice session, LSJ completed 32 practice trials in which she played the material on the viola from the sheet music. The practice sessions were designed to model how a musician might rehearse a novel piece of music: the material was played at increasing tempos across a practice session, and included short segments as well as the whole piece.

In nine of the practice trials in each session, LSJ played the piece in its entirety at varying tempos controlled with a metronome: once at 96 bpm, once at 108 bpm, four times at 120 bpm, and three times at 144 bpm. Interspersed with the full-piece practice trials were 23 practice trials in which LSJ played short (4–9 bar) pre-specified segments of the piece at the different tempos. Each practice session lasted approximately an hour (62 and 69 min for sessions 1 and 2, respectively).

Test trials, in which LSJ played all three pieces in their entirety at 144 bpm, were administered immediately before and after each practice session and once again after a 14-day delay.

In all practice and test trials, LSJ was presented with sheet music and instructed to play it to the best of her ability, without interruption, and in time with the metronome. Before beginning, she was asked to play an unrelated piece from sheet music to warm-up. A professionally trained musician tuned LSJ’s viola before each practice session.

On each of her many encounters with a piece, LSJ showed no awareness of having seen the piece before. In fact, she made the same humorous remark related to the pieces’ title nearly every time she was presented with the sheet music, suggesting that she had no recollection either of having seen the sheet music before or of having made the same comment about it. Although LSJ generally performed the music willingly and seemed to enjoy playing, occasionally she expressed her discontent with being asked to play such difficult new material at a tempo she felt was too fast. She repeatedly indicated that she was playing the material for the very first time, even on the last test trials after repeated performances of all pieces. On these occasions, she was encouraged to do the best she could.

Since the time of her illness, LSJ has played the viola only occasionally. In the months prior to our study, she played for short periods several times per week, in the company of a family member.

#### Analyses

LSJ’s test trial performance was evaluated via two methods: note-by-note analyses and subjective performance ratings by experienced string players.

##### Note-by-note analyses

Two independent coders counted the number of individual notes LSJ played correctly with respect to pitch, relative rhythm, note duration, and metronome-dictated tempo. One point was awarded for every correctly played note, and zero points were given for notes in which any of the above aspects were incorrect. In pitch, notes were allowed to deviate from the written notation by one half of a semitone or less to be considered correct. In rhythm and tempo, notes played slightly ahead or behind the beat were scored as correct, but those more than a half-beat off or in a clearly wrong rhythmic pattern or duration were scored as incorrect.

One exception was made to the general scoring rule. As meter is temporally and hierarchically organized, a single rhythmic error can cause all subsequent notes to align incorrectly to the originally established meter, yielding a score of zero for all notes afterward. This occurs, for example, every time the performer misses or skips a beat, or plays an extraneous note. To avoid penalizing all consecutive notes because of a single error, coders identified the first run of four consecutive correctly played notes after such errors. This four-note run was used to establish a new meter with respect to the metronome, and coding was resumed from (and including) this four-note string. These four notes had to be correct in both pitch and rhythm and in synchrony with the metronome.

The coders were both skilled amateur musicians. The first coder was one of the authors (Jussi Valtonen), who was blind to test trial, but not to which pieces had been practiced. The second scorer was blind to both test trial and practiced pieces, and was otherwise not involved with the study. For all test trial performances, the two coders scored all notes in order from audio recordings of intact whole performances. The performances were scored in three blocks, with all test trials for one piece in one block. The order of the blocks and the order of test trial performances within each block were randomized for each of the two coders. Mean inter-rater reliability was 0.88 (0.87, 0.86, and 0.90 for Pieces A, B, and C, respectively). All discrepancies in coding were discussed and resolved between the two coders, and the resolved scorings were used for final analyses.

##### Subjective performance ratings

Subjective performance ratings were collected from six string instrumentalists, all blind to both test trial and to which pieces had been practiced. All raters were professional musicians or music students who had either the viola (*n* = 4) or the violin (*n* = 2) as their main instrument (mean number of years played 13.7; range 9–22). The raters were recruited from the Johns Hopkins Peabody Conservatory, where they pursued or had completed an undergraduate or graduate degree in viola or violin performance or had music as a minor subject. All raters reported prior experience in evaluating musical performances, either through formal music training, through teaching, or both.

The raters evaluated all LSJ’s test trial performances on a 1–5 scale according to four qualitative dimensions of musical performance. Based on previous research (Zdzinski and Barnes, 2002), we chose three dimensions that form separate factors in string performance ratings: (1) *intonation*, reflecting pitch accuracy and the degree to which the pitches sound correct in context (1 = most pitches are out of tune, 5 = virtually all pitches are accurate, with virtually no adjustments needed to fix them), (2) *rhythm*, reflecting the rhythmic accuracy of executed patterns and how accurately they match the sheet music (1 = most rhythmic patterns are incorrect, 5 = virtually all rhythmic patterns are accurate), and (3) *tone*, reflecting the quality of sound in the played notes (1 = sound is unfocused, making it difficult to discern many notes, 5 = sound is clear, focused, and warm virtually throughout). In addition, the musicians were instructed to evaluate the performance (4) *overall*, taking into account all relevant aspects of skilled musical performance (values 1–5 were left for the rater to specify).

Before evaluating LSJ’s performances, the raters were given the sheet music and familiarized themselves with the pieces by playing them on their own instrument. They also heard computer performances of each piece. Because the exact criteria and degree of precision required for an accurate performance are a matter of subjective opinion, and the relative weight given to expressive nuances will vary among raters, we attempted to calibrate different rating expectations before evaluations of test trials. To this end, the raters heard a recording of an error-free performance by LSJ of an unrelated song that she knows well. The raters were instructed to consider this performance as qualifying for a rating of 5 on the five-point scale.

Audio recordings of LSJ’s test trial performances were presented to each rater in three blocks, with all test trials of one piece in one block. The order of blocks and the order of trials within each block were randomized across raters.

All raters provided written informed consent. LSJ provided oral assent, and her legal guardian provided written consent for her. The study protocol was approved by the Homewood Institutional Review Board at Johns Hopkins University.

## Results

### Note-by-note scores

Note-by-note scorings of LSJ’s viola performances showed that as expected, all three pieces were initially challenging for her to sight-read at the designated tempo. In her first test trial performances, before any pieces had been practiced, the mean percentage of correctly played notes across the three pieces was 29%, showing that the complexity of the sight-reading material clearly exceeded her capabilities at the requested tempo. Qualitatively, her performances in all trials included several temporal breakdowns and violations of the underlying beat, demonstrating that she was unable to maintain the expected temporal continuity in performance.

As all pieces were performed in five test trials, some improvement could potentially be expected to occur overall, merely as a function of repeated test trial performances and regardless of piece type. Learning effects resulting from training on Pieces A and B should be revealed by greater improvement for those pieces than for the unpracticed Piece C. In particular, we expected learning effects to be revealed by a positive linear trend that is larger for practiced than unpracticed pieces. In addition, we might also expect to see a quadratic trend, reflecting a plateauing of scores from Test Trial 4 to Test Trial 5, as no additional training took place over the delay.

To investigate potential learning in LSJ’s performance, we compared three critical trials: Test Trial 1, administered before any pieces had been practiced, Test Trial 4, administered on the same day after both target pieces had been practiced, and Test Trial 5, administered 14 days after practice (see Figure 2). Because potential learning effects were expected to be similar for both practiced pieces, the data were collapsed across Pieces A and B and compared to the unpracticed Piece C.

**Figure 3:**
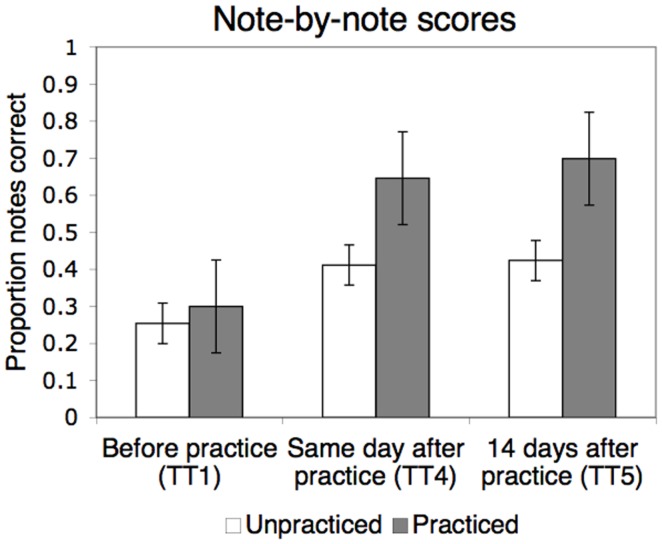
**Mean proportion of correctly played notes in LSJ’s sight-reading performances on the viola before practice, after practice on the same day, and after a 14-day delay (Test Trials 1, 4, and 5, respectively)**. Results have been collapsed across the two practiced pieces, Piece A and Piece B. Error bars represent standard error of the mean.

As shown in Figure 3, the targeted learning effects can be seen very clearly in these trials. Overall, the mean percentages of correct notes increased across pieces from 29% in Test Trial 1 to 61% in Test Trial 5. A repeated-measures ANOVA (2 piece types × 3 test trials) showed a significant main effect of piece type [practiced versus unpracticed; *F*(1,246) = 71.23, *p* < 0.001], a significant main effect of test trial [*F*(2,492) = 111.95, *p* < 0.001], and a significant interaction [*F*(2,492) = 19.58, *p* < 0.001]. For the main effect of test trial, there was a significant linear trend across trials [*F*(1,246) = 161.38, *p* < 0.001], reflecting an overall improvement in note scores from Test Trial 1 to 5. The quadratic trend was also significant [*F*(1,246) = 44.06, *p* < 0.001], reflecting the increase in note scores immediately after practice (between Test Trials 1 and 4) followed by plateauing across the 14-day delay (from Test Trial 4 to 5).

Critically, the improvement in LSJ’s performance was more pronounced in the practiced pieces relative to the unpracticed piece in both the linear and the quadratic trends, *F*(1,246) = 43.66, *p* < 0.001 and *F*(1,246) = 3.95, *p* < 0.05, respectively. As can be seen in Figure 3, note scores improved after practice both for unpracticed and practiced pieces, but this improvement was larger for the practiced pieces. The learning was also retained during the 14-day delay: as shown in the figure, the note scores for Test Trial 5 stayed almost exactly at the level of Test Trial 4 for both practiced and unpracticed pieces, but were higher for practiced pieces (mean scores 70 and 43% for practiced and unpracticed pieces, respectively, in Test Trial 5). The scores for practiced and unpracticed pieces improved by 40 versus 17 percentage points from Test Trial 1 to 5, respectively.

The learning effects observed in the analyses are also apparent when we examine the results for the individual pieces on each test trial (see Table 5). After each practice session, the piece practiced in that session showed the largest performance improvement. After Session 1, in which Piece A was practiced, the note-by-note score improved by 27 percentage points for piece A, versus 18 and 19 percentage points for pieces B and C, respectively. After Session 2, in which Piece B was practiced, Piece B showed a 36 percentage-point improvement, versus a 24 percentage-point improvement for Piece A and a 6 percentage-point decline for Piece C. After a piece was practiced, performance on that piece remained relatively stable. For example, the note score for Piece A immediately after practice was 66%, and remained high (76%) 2 weeks later. Similarly, the score for Piece B was 70% immediately after practice, and 64% after 2 weeks. The sole exception to this pattern was LSJ’s poor score (35%) for Piece A on Test Trial 3 (the Session 2 pre-test immediately prior to practice on Piece B). On this particular trial, LSJ decided, either deliberately or by accident, to play most of the piece in half tempo, yielding a score of zero for all the corresponding notes. On the remaining test trials (Trials 4 and 5), her performance on Piece A was again much better (for a figure showing note scores on all three pieces in all test trials, see Supplementary Material).

**Table 5:** **Mean percentages of notes according to piece and test trial**.

Test trial	Piece A correct (%)	Piece B correct (%)	Piece C correct (%)
1. Before practice	39	21	26
2. After practicing A	66	39	45
3. Before practicing B	35	34	47
4. After practicing B	59	70	41
5. After 14-day delay	76	64	43

*Piece A was practiced between trials 1 and 2, and Piece B between trials 3 and 4. Piece C was not practiced*.

### Subjective performance ratings

**Figure 4:**
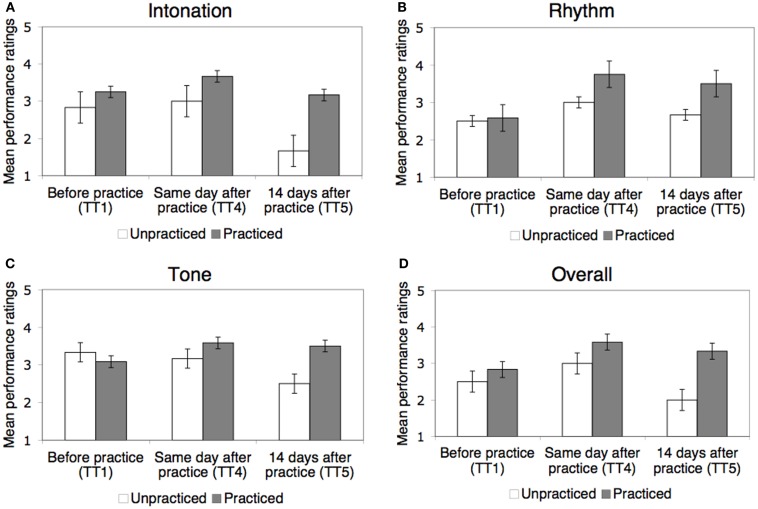
**LSJ’s music performance on practiced and unpracticed pieces before and after practice on the same day and after a 14-day delay (Test Trials 1, 4, and 5, respectively) as evaluated by experienced string players**. Performance was evaluated on a 1–5 scale separately for intonation **(A)**, rhythm **(B)**, tone **(C)**, and overall **(D)**. Results were collapsed across the two practiced pieces, Piece A and Piece B. Error bars represent standard errors of the mean.

Learning of the practiced pieces was also evident in the subjective performance ratings by string players (Figure 4). Mean ratings of overall musical performance increased across all pieces from 2.72 before practice to 3.39 immediately after practice, and ratings of intonation, rhythm, and tone from 3.11 to 3.44, from 2.56 to 3.50, and from 3.17 to 3.44, respectively. As illustrated in Figure 4, effects of learning for the practiced pieces can be seen in the ratings in all four dimensions.

As with note-by-note scores, the results were analyzed for the critical three test trials most comparable to each other, Test Trials 1, 4, and 5, with the data collapsed across the two practiced pieces. Separate repeated-measures ANOVAs (2 piece types × 3 test trials) revealed a significant main effect of test trial in all rating dimensions: intonation [*F*(2,10) = 9.07, *p* < 0.01], rhythm [*F*(2,10) = 6.52, *p* < 0.05], tone [*F*(2,10) = 6.49, *p* < 0.05], and overall ratings [*F*(2,10) = 8.72, *p* < 0.01], showing that performance ratings improved across test trials. The main effect of piece type (practiced versus unpracticed) was also significant for intonation [*F*(1,5) = 17.1, *p* < 0.01], tone [*F*(1,5) = 7.66, p < 0.05] and overall ratings [*F*(1,5) = 13.35, *p* < 0.05].

Most importantly, the interaction between piece type and test trial was significant in ratings of intonation [*F*(2,10) = 6.150, *p* < 0.05] and tone [*F*(2,10) = 10.181, *p* < 0.01], showing that practice had affected the ratings differently in the practiced pieces relative to the unpracticed piece in these dimensions. The interaction also approached significance in overall ratings [*F*(2,10) = 3.545, *p* = 0.069], but was not significant in rhythm ratings [*F*(2,10) = 1.746, *p* = 0.224]. For intonation and tone, ratings for the practiced pieces improved after practice and stayed the same or slightly declined over the 14-day delay, whereas ratings for the unpracticed piece showed less or no improvement and a marked decline over the delay [*F*(1,5) = 9.494, *p* < 0.05 and *F*(1,5) = 16.304, *p* < 0.01 for linear trend in practiced versus unpracticed pieces in intonation and tone ratings, respectively].

The pattern revealed by these analyses can also be seen in the ratings for the individual pieces on each test trial (see Table 6). After each practice session, the practiced piece showed the greatest improvement in ratings on each dimension, and thereafter showed better performance than the unpracticed piece.

**Table 6:** **Mean performance ratings for all evaluated performance dimensions according to piece and test trial**.

Performance dimension	Test trial	Piece A	Piece B	Piece C
Intonation	1	2.83	3.67	2.83
	2	3.33	3.00	2.50
	3	3.33	2.67	2.67
	4	3.17	4.17	3.00
	5	3.33	3.00	1.67
Rhythm	1	2.83	2.33	2.50
	2	4.00	2.83	2.33
	3	2.83	2.17	2.67
	4	3.67	3.83	3.00
	5	4.33	2.67	2.67
Tone	1	3.17	3.00	3.33
	2	3.67	2.83	2.50
	3	3.50	2.83	2.67
	4	3.17	4.00	3.17
	5	3.83	3.17	2.50
Overall	1	2.83	2.83	2.50
	2	3.83	3.00	2.67
	3	3.17	2.17	2.67
	4	3.17	4.00	3.00
	5	3.67	3.00	2.00

*Piece A was practiced between trials 1 and 2, and Piece B between trials 3 and 4. Piece C was not practiced*.

## Discussion

We examined the learning of novel pieces of viola music by a newly identified amnesic patient who has bilateral MTL damage involving near-complete destruction of the hippocampus. Despite her extreme anterograde amnesia and lack of recollection of having played the pieces, LSJ’s performance improved for two pieces after practice relative to an unpracticed control piece^2^. These performance improvements were evident both in detailed note-by-note analyses, and in string instrumentalists’ subjective ratings of whole-piece performances. Moreover, learning was apparent not only on the day of practice but also 14 days later. As LSJ has virtually no remaining hippocampal tissue, these results show that learning to perform new music can occur in the absence of the hippocampus. Although two previous studies have investigated music learning in patients with MTL damage, the underspecification of the patients’ hippocampal damage makes the implications unclear, as it is impossible to rule out the contribution of remaining hippocampal tissue to learning. To our knowledge, our study is the first demonstration that non-hippocampal structures alone can support learning for music performance through sight-reading.

That such a complex learning process is possible without the hippocampus is somewhat surprising, given that the hippocampus is essential for memory functions that likely contribute to the learning process in neurologically intact musicians. For example, prior research has shown that the hippocampus is critical for both single item and associative declarative memory (Squire et al., 2004), and is engaged during the implicit learning of temporal-motor sequences (Schendan et al., 2003; Robertson, 2007; Gheysen et al., 2010). While the hippocampus is important in the normal process through which music is learned, our results suggest that non-hippocampal structures also can play a critical role.

The contrast between the learning observed in this study and LSJ’s learning impairments in other tasks is remarkable: for example, LSJ fails to show statistical learning (Schapiro et al., 2014), which occurs largely automatically and implicitly in healthy adults (Kim et al., 2009). In the three experiments conducted by Schapiro et al. (2014), LSJ was passively exposed to visual shapes, spoken syllables, visual scenes, or auditory tones in sequences that contained temporal regularities. In contrast to control participants, she showed no ability to detect the regularities in any of the sequences. In addition, with regard to declarative memory, LSJ’s ability to acquire new information is minimal and requires massive training. When trained on six commercial logos and their corresponding company names and product categories, LSJ showed very little learning after 114 practice sessions, totaling nearly 30 h of training (Gregory et al., 2013). In comparison, the learning effects observed in the current study were achieved rapidly, with only an hour of practice for each of the two pieces. On the other hand, however, LSJ’s implicit learning abilities are not generally preserved for all music-related tasks: LSJ is also impaired at the MBEA incidental memory test (Peretz et al., 2003), a yes/no recognition task that probes familiarity for melodies presented in earlier tasks of the assessment battery. This finding, from a task that is arguably much simpler than the one used in the current study, suggests that LSJ’s profound learning deficits extend also to measures of implicit learning in music perception, and stands in contrast to the learning seen in the current music performance experiment.

LSJ’s learning of new musical pieces may be possible in part because musical performance is likely to draw on many different cognitive functions that may be supported by non-hippocampal structures, including those that were left intact after her illness. For example, learning new musical pieces might engage implicit learning mechanisms, which have been argued to be distributed across different brain regions (Reber, 2013)^3^. Playing an instrument requires processing information simultaneously from the visual, auditory, and tactile modalities and from sensory organs in muscles, tendons, joints, and skin (Sloboda, 1984; Palmer, 1997, 2006; Altenmüller and Schneider, 2009; Chaffin et al., 2009). The correct note sequences have to be executed in concert with emotional processing and considerations for esthetic and musical expression. Neurally, areas that could support these functions are likely to encompass much of the brain, ranging from regions related to basic visual object recognition and sensorimotor functions to several separate pathways projecting from the primary auditory cortices to various targets (Peretz and Zatorre, 2005; Zatorre et al., 2007; Altenmüller and Schneider, 2009). With regard to specific aspects of music performance, several cortical and sub-cortical regions have been implicated in prior research. For example, controlling timing has been linked to the cerebellum, basal ganglia, and the supplementary motor area, while controlling aspects of rhythm has been connected to the dorsal premotor cortex, lateral cerebellar hemispheres, and prefrontal cortex. The inferior frontal regions have been implicated with retrieval processes from long-term representations [for reviews, see Janata and Grafton (2003), Peretz and Zatorre (2005), Zatorre et al. (2007), and Levitin and Tirovolas (2009)]. Conceivably, the recruitment of such a wide range of interconnected cortical and sub-cortical neural functions could support LSJ’s new learning of musical pieces. Moreover, while virtually all of LSJ’s hippocampal tissue has been destroyed, some tissue remains in her MTL (i.e., 40–60% of tissue in parahippocampal, entorhinal, and perirhinal cortices), and it is possible that this remaining tissue is contributing to learning.

LSJ not only learned new musical pieces within the practice sessions but also showed retention of the learning over a 14-day period. This result raises the question of how long-term memories can be preserved in the absence of the hippocampus. Some types of implicit learning can be retained for remarkably long time periods even in amnesic patients, including those with hippocampal damage (Gabrieli et al., 1993; Hayman et al., 1993; Hamann and Squire, 1995; Corkin, 2002). However, previous work has also indicated that the hippocampus is important for how declarative memories are stabilized or otherwise remain accessible over time (Nadel and Moscovitch, 1997; Squire et al., 2004; Bontempi and Frankland, 2005; Nadel and Peterson, 2013), and that the hippocampus is involved in memory consolidation for implicitly learned temporal-motor sequences (Albouy et al., 2008, 2013a,b). Our results suggest that structures outside of the hippocampus can support at least some of the processes by which memory representations for music performance are retained over time. Future research will be needed to shed light on how this occurs.

While music performance has at times been described as a case of “procedural,” “non-declarative,” or “motor” learning, we argued earlier that the learning of new musical pieces for performance is likely to go beyond what Stanley and Krakauer (2013) refer to as improved motor acuity. The current study and its results would also seem to support this suggestion. For one, the pieces did not pose novel motor acuity challenges for LSJ – for example, through exceptionally inconvenient fingerings or atypical position shifts – but rather asked her to carry out well-learned movements associated with familiar notes (although in novel sequences). In contrast, the pieces did pose cognitive challenges: even after practice, there were frequent occurrences of temporal discontinuities such as interruptions, temporal breakdowns, pauses, hesitations, and violations of the underlying beat. As Drake and Palmer (2000) point out, pauses and other forms of temporal disruption are considered a measure of cognitive load, both in music production and in other complex sequence planning tasks such as speech. Thus, there was considerable room for improvement in LSJ’s ability to cope with the excessive cognitive load, and this – and not motor acuity – is likely where her learning occurred.

It is, of course, possible that LSJ also improved in motor acuity (for example, in the sequence-specific transitions between adjacent notes in these unique compositions), but this was not measured by our methods. Our methods focused on her acquisition of note accuracy, intonation, rhythm, and tone, and measured her ability to process written notation in order to execute the corresponding motor responses. In the note scores, notes counted as correct were as far as halfway between the correct and the adjacent pitch and therefore, notes counted as incorrect were very clearly off target or parts of long sequences she was not able to attempt to play at all. Her scores for correct notes increased from 30 to 70% on the practiced pieces, an improvement of more than 130%. Improved motor acuity may perhaps have helped move some very incorrectly executed notes to the zone of roughly correct, but it seems highly unlikely that such a large learning effect could be completely accounted for this way.

Many questions remain for future research concerning the specific cognitive and neural mechanisms underlying learning for music performance in LSJ and neurologically intact individuals. First, the current experiment did not assess one, specific cognitive ability, or even a collection of them that could be precisely specified. While many of the cognitive processes that underlie music performance have been identified in behavioral studies (e.g., Sloboda, 1985; Palmer, 2006), more theoretical and empirical work is needed to fully decompose the cognitive processes involved and their neural substrates. Second, we can ask whether neurologically intact violists with similar expertize would have shown more learning with the same amount of (non-standard) structured practice. Such research would speak regarding which aspects of learning can be supported by non-hippocampal structures and provide further ways to delineate the links between the cognitive and neural aspects of music performance. Third, a direct comparison of a hippocampal amnesic’s performances in non-musical motor learning (e.g., the SRT task) and in music performance would shed more light both on the role of the hippocampus in different contexts of complex motor learning and on the different cognitive-motor processes involved in these tasks. In addition, some authors have argued that while acquiring conscious memories of the learning episode depends on the hippocampus, the subjective feeling of familiarity is supported by perirhinal cortex (Corkin, 2002). LSJ’s apparent lack of awareness that the same musical material was being presented repeatedly in the practice and test trials suggests that she did not acquire a feeling of familiarity for the pieces, but it would have been interesting to collect familiarity judgments from her over the course of the study.

## Conflict of Interest Statement

The authors declare that the research was conducted in the absence of any commercial or financial relationships that could be construed as a potential conflict of interest.

## Supplementary Material

The Supplementary Material for this article can be found online at http://www.frontiersin.org/Journal/10.3389/fnhum.2014.00694/abstract.

## Footnotes

^1^For two other related neuropsychological studies of music learning in which the role of the hippocampus was not investigated, see Fornazzari et al. (2006) and Baur et al. (2000).

^2^It should be noted that note scores also improved for the unpracticed piece, albeit less markedly than for the pieces targeted for practice. As LSJ was exposed to the unpracticed control piece five times in the course of the experiment, the results may reflect improvement through the same learning mechanism as with the practiced pieces, merely to a lesser extent.

^3^It is important to note that the broad term implicit learning can refer to many distinct processes. While many amnesic patients with hippocampal damage have shown normal or near-normal learning in several implicit learning and implicit memory tasks (Corkin, 1968; Reber and Squire, 1994; Stefanacci et al., 2000; Verfaelllie et al., 2012; Eichenbaum, 2013; Reber, 2013), the evidence is mixed. Implicit learning is not always intact in amnesic patients (Cohen et al., 1997; Curran, 1997; Channon et al., 2002), and at least some forms of implicit learning do seem to depend on the hippocampus (Chun and Phelps, 1999; Squire and Knowlton, 2000). Determining the precise contribution of the hippocampus for implicit learning processes is difficult because measures of implicit learning often also recruit explicit memory processes (Gabrieli, 1998). An exhaustive review of these findings is beyond the scope of this paper, as our goal was to investigate whether the hippocampus is necessary for the learning of new music.
