# Supplementary material for: New Learning of Music after Bilateral Medial Temporal Lobe Damage: Evidence from an Amnesic Patient
Source: Front Hum Neurosci. 2014 Sep 3;8:694. doi: 10.3389/fnhum.2014.00694 (PMC4153029; doi:10.3389/fnhum.2014.00694)
Supplement: Supplementary file 1 [file Presentation_1.ZIP › Supp Mat B.PDF]

Piece B

Score

JJR

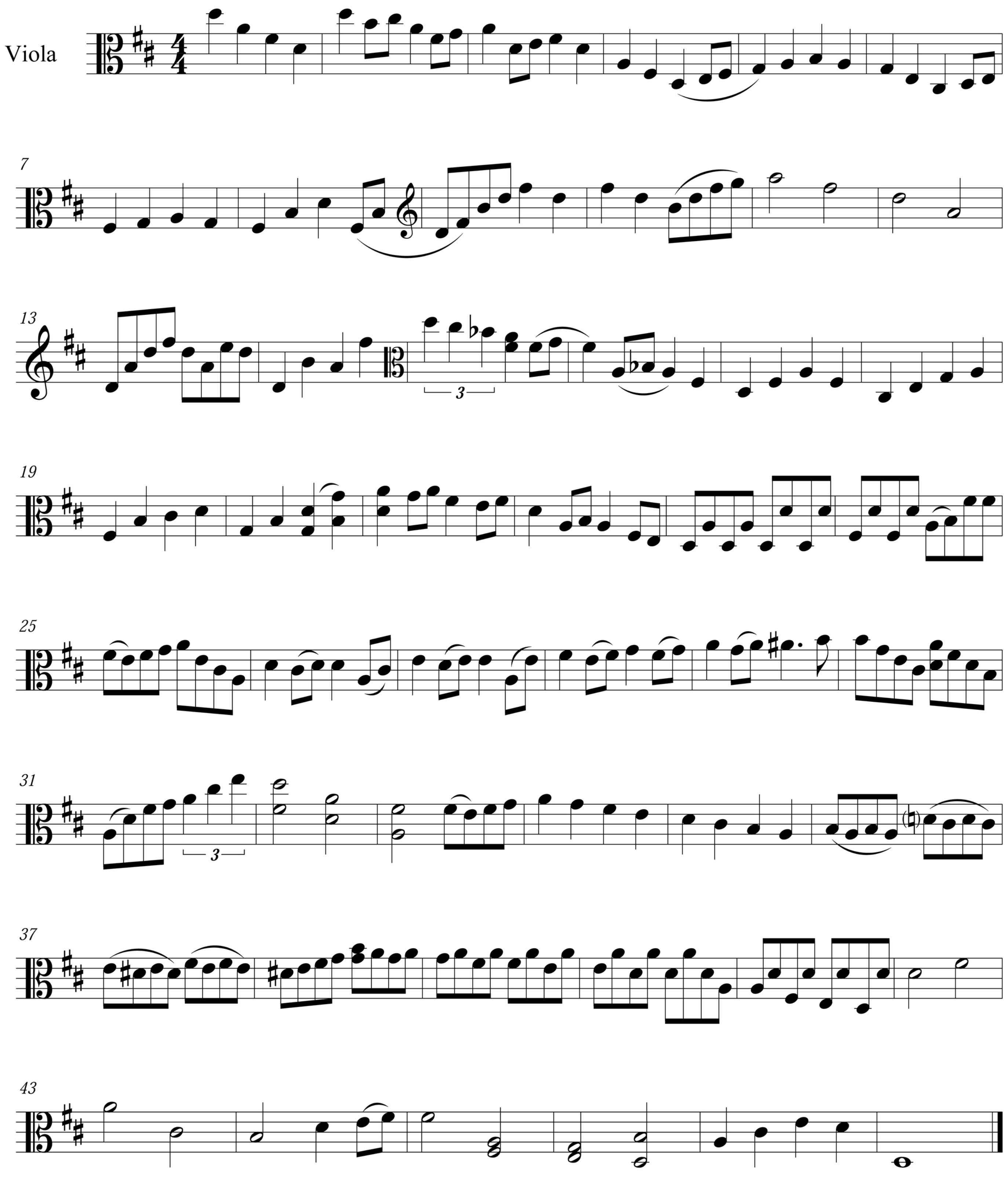

©2012
