# Supplementary material for: New Learning of Music after Bilateral Medial Temporal Lobe Damage: Evidence from an Amnesic Patient
Source: Front Hum Neurosci. 2014 Sep 3;8:694. doi: 10.3389/fnhum.2014.00694 (PMC4153029; doi:10.3389/fnhum.2014.00694)
Supplement: Supplementary file 1 [file Presentation_1.ZIP › Supp Mat captions.PDF]

Supplementary Material

New Learning of Music after Bilateral Medial Temporal Lobe Damage: Evidence from an Amnesic Patient

Jussi Valtonen[1]*, Emma Gregory[2], Barbara Landau[2] and Michael McCloskey[2]

[1]Institute of Behavioural Sciences, University of Helsinki, Helsinki, Finland
[2]Department of Cognitive Science, Johns Hopkins University, Baltimore, Maryland, USA

*** Correspondence:** Jussi Valtonen, Institute of Behavioural Sciences, University of Helsinki, P.O. Box 9, Helsinki, FI-00014, Finland.
jussi.valtonen@helsinki.fi

1. Supplementary Data

1.1. Computer software performances of the pieces used in the study.

Supplementary Audio File 1. Computer performance of Piece A.

Supplementary Audio File 2. Computer performance of Piece B.

Supplementary Audio File 3. Computer performance of Piece C.

1.2. Sheet music for the pieces used in the study.

Supplementary Data Sheet 1. Sheet music for Piece A.

Supplementary Data Sheet 2. Sheet music for Piece B.

Supplementary Data Sheet 3. Sheet music for Piece C.
